# Medical Library Association Diversity and Inclusion Task Force 2019 Survey Report

**DOI:** 10.5195/jmla.2020.948

**Published:** 2020-07-01

**Authors:** JJ Pionke

**Affiliations:** 1 pionke@illinois.edu, Assistant Professor, University Library, University of Illinois–Urbana-Champaign

## Abstract

**Objective:**

The goal of this survey by the Medical Library Association (MLA) Diversity and Inclusion Task Force was to have a better understanding of the demographics of the association as well as ascertain how the membership feels about MLA's diversity efforts.

**Methods:**

A survey was created with the input of both task force members as well as MLA professional staff. It was administered via SurveyMonkey and distributed through email over the course of two weeks in October 2019.

**Results:**

The demographics portion of the survey—beyond asking the usual questions about race or ethnicity (72% white), age (65% between 30 and 59), and so on—also asked questions that were more specific to diversity including, but not limited to, gender representation (79% female), sexuality (67% heterosexual), military service (97% have never served), ability (26% have anxiety sometimes or in certain situations), and college financial aid (49% used federal student loans). Diversity-specific questions asked about diversity, equity, and inclusion (DEI) in the association: 59% strongly agreed or agreed that MLA has a strong commitment to DEI; 54% felt that the amount of time that association was spending on DEI issues was just about right; and 56% were very satisfied or satisfied with the DEI environment at MLA. Members also reported feeling like they belonged in MLA (59%), they were treated with respect (77%), and they were valued by MLA (59%)

**Conclusion:**

The survey paints a picture of the membership that is much deeper than any previously conducted membership survey. It shows the diversity of membership, especially in terms of ability and religion. Generally, the membership feels that MLA is right on target with the level of focus that MLA is giving issues of diversity. This survey reinforces the diversity work that has been done and supports diversity work in MLA in the future.

## EXECUTIVE SUMMARY

In fall 2019, the Diversity and Inclusion Task Force administered an online survey to the membership of the Medical Library Association (MLA) to ascertain not only the demographic makeup of the association, but also to have a better understanding of how the membership felt about MLA itself, as well as the diversity, equity, and inclusion (DEI) efforts of the association. Because respondents did not necessarily answer every question, the data in this report are promulgated as percentages rather than as raw numbers.

In terms of survey respondents' attitudes about the association's efforts regarding DEI, the results were largely positive:

- 59% strongly agreed or agreed that MLA has a strong commitment to DEI
- 54% felt that the amount of time that the association is spending on DEI issues is just right
- 56% were very satisfied or satisfied regarding the DEI environment in MLA

Respondents were also asked how they felt about MLA as an association and their places in it.

- 26% have been members of MLA for less than 4 years; 17% have been members of MLA for 25 years or more
- 50% paid their own annual membership fees
- 59% strongly agreed or agreed that they felt valued as members of MLA
- 59% strongly agreed or agreed that they felt a sense of belonging in MLA
- 77% strongly agreed or agreed that they were treated with respect in MLA
- 64% strongly agreed or agreed with the statement, “I feel welcomed and included in MLA Annual Meetings.”
- 76% strongly agreed or agreed that they were able to participate in MLA at the level that they desire
- 69% strongly agreed or agreed that they were able to find at least one community or group in MLA in which to belong
- 76% strongly agreed or agreed that being a member of MLA has had a positive effect on their professional growth

As a representation of MLA membership, survey respondents generally demographically corresponded with the relative norms seen across librarianship as a profession.

- 65% were between the ages of 30 and 59
- 72% were white
- 95% lived and worked or went to school in the United States
- 97% had never served in any branch of the US military
- 79% were female
- 67% were heterosexual
- 26% stated that they had anxiety sometimes or in certain situations
- 42% were Christian
- 77% were caregivers in some capacity
- 92% had master's degrees in library and information science
- 49% used federal student loans to pay for their education
- 21% were categorized as medical librarians or librarians
- 39% earned between $50,000 and $70,999

There were some surprises in the data. For instance, Christians represented the largest group in the association at 42%, but as a group they were under 50% of the membership. Likewise, the data for the question that asked about ability was very surprising in that the data reflected far more people with disabilities than had ever been recorded in a library association. Generally speaking, the membership was happy with the efforts of the association to be more diverse, equitable, and inclusive. The membership also generally felt respected and included in the association. Respondents had many suggestions across the questions where commenting was available, which indicated involvement and interest in the goings on of the association by the membership. There is, of course, always room for improvement, and data from this survey will be examined closely by association leadership.

## INTRODUCTION

In fall 2019, MLA determined that the association's Diversity and Inclusion Task Force would complete an online survey of the membership with the purpose of better understanding not only the demographic makeup of the association, but also how members felt about the association's DEI efforts, as well as how they felt about the association itself and their position in it.

## METHODOLOGY

A subcommittee of the whole was formed to develop the questions. Working with MLA's resident survey expert, Kate Corcoran, questions were formulated by members of the task force, tested, and then loaded into SurveyMonkey. The survey was active from October 15, 2019–October 31, 2019, and was sent to 2,691 email addresses. Nine hundred eighteen people answered the first question, which equals a response rate of 34%. Several rounds of emails were sent to the membership over that period to encourage participation. Because not all respondents answered all questions, percentages are used throughout this report rather than raw data.

## RESULTS

### Diversity, equity, and inclusion

One of the primary purposes of the survey was to find out what the membership thought about the association's DEI efforts that have been ongoing for the last several years. To that end, the questions in this section were designed to ascertain how well MLA is perceived to be doing regarding DEI efforts.

#### Commitment to diversity, equity, and inclusion.

Respondents were asked to what degree they felt that MLA had a commitment to DEI. Forty-four percent stated that they agreed that MLA has a strong commitment, with 59% agreeing or strongly agreeing with the statement. Only 8% strongly disagreed or disagreed with the statement, and 33% neither agreed nor disagreed.

#### Emphasis on issues of diversity, equity, and inclusion.

Another question asked respondents to what degree they felt that MLA focused on issues of DEI. Fifty-four percent felt that the amount of time spent on these issues was “about right”; 37% felt that MLA is spending “too little” or “far too little” time and energy on DEI issues; and 8% felt that MLA is spending “too much” or “far too much” time on these topics.

Respondents also had an opportunity to leave comments on this question. There were 197 comments, of which 82 were negative and reflected the mentality that MLA is either doing too much or not enough. For instance, one respondent stated that:

“I think MLA has jumped on the diversity and inclusion bandwagon without considering whether it's pertinent to our organization. We can embrace the goals without spending our limited time and resources on something that in my opinion is peripheral to the mission of the Medical Library Association.”

In contrast, there were seventy positive comments, of which the majority reflected that MLA was doing well and that there was more to be done. For example:

“At first I thought the recent emphasis was too much. Then I realized that was probably because I'm part of a privileged group and it's time to get over it.”

The forty-five neutral comments ran the gamut, ranging from “no opinion” to listing suggestions for improvement in the organization, at MLA annual meetings, and so on.

#### Satisfaction with the environment of MLA in relation to diversity, equity, and inclusion.

Respondents were asked to rate their satisfaction with MLA's environment regarding DEI since becoming a member of MLA. Fifty-six percent were either satisfied or very satisfied; only 10% were very dissatisfied or dissatisfied; and 34% were neither satisfied nor dissatisfied.

### The Medical Library Association (MLA)

Several questions pertained specifically to individuals' relationships with the association, such as how long respondents had been members of MLA as well as whether the respondents felt valued in MLA.

#### Length of MLA membership.

**Figure 1:**
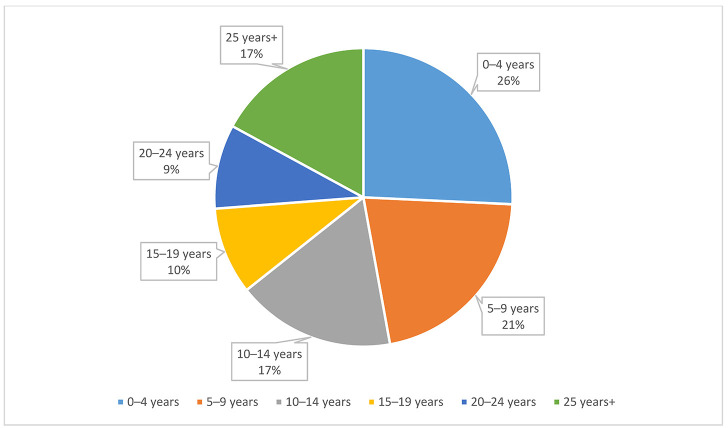
Length of Medical Library Association (MLA) membership by percentage

When asked how long the member had been part of the association, the largest percentage was in the 0–4 years range, at 26%. There was also a fairly significant percentage at the completely opposite end of the spectrum, with 17% of respondents having been members for 25 years or more. Figure 1 shows a further break down.

#### Annual membership.

Survey takers were asked whether or not their employer paid for their annual MLA membership. Fifty percent responded that their employer did not pay for their annual MLA membership; 27% selected “Yes, full membership outside of professional development funds”; 13%: “Yes, full membership if I use professional development money”; and 6%: “Yes, part of my membership.” There were 35 comments with this question, of which the majority elaborated on how their memberships have been paid, or not, in the past.

#### Value as an individual in MLA.

Forty-five percent of respondents agreed that they felt valued as individuals in MLA; 59% strongly agreed or agreed that they felt valued in MLA. Only 7% disagreed or strongly disagreed, and 34% neither agreed nor disagreed.

#### Belonging in MLA.

Respondents were asked to what degree they felt that they belonged in MLA. Fifty-nine percent strongly agreed or agreed that they felt they belonged. Only 11% strongly disagreed or disagreed that they belong, and 30% neither agreed nor disagreed.

#### Treatment with respect in MLA.

Respondents were asked whether they felt that they were treated with respect in MLA. Fifty-six percent agreed that they were treated with respect; 77% strongly agreed or agreed that they were treated with respect in MLA. Only 4% strongly disagreed or disagreed, and 19% neither agreed nor disagreed.

There was a follow up question for those who felt that they were not treated with respect in MLA with a total of nineteen comments. A variety of issues were raised, including but not limited to, cliquishness in the organization, prohibitive costs, lack of diversity, and ageism (both older to younger and younger to older).

#### Welcome and inclusion at MLA annual meetings.

Respondents were asked to indicate to what degree they agreed with the statement: “I have felt welcomed and included in MLA annual meetings.” Sixty-four percent strongly agreed or agreed; 6% strongly disagreed or disagreed; and 16% neither agreed nor disagreed.

Respondents were able to leave a comment if they indicated that they felt unwelcome or excluded when they attended MLA annual meetings. There were thirty-six comments, which included topics ranging from MLA annual meetings being cliquish, to the annual meeting being too expensive to attend, to difficulty connecting to others because of the way the annual meeting was structured, and other factors.

#### Participation level in MLA.

Another question inquired whether or not respondents were able to participate in MLA at the level that they desired. Seventy-six percent strongly agreed or agreed that they were able to participate at the level that they wanted to; 8% strongly disagreed or disagreed; and 16% neither agreed nor disagreed.

Respondents were able to follow up negative responses with comments about why they felt they had not been able to participate in MLA to the degree to which they wanted to. There were forty-nine comments in total, with similar answers to the previous question. Respondents commented about cliquishness, cost, and communication problems between the association and the membership.

#### MLA communities or groups.

Respondents were asked whether they had been able to find 1 or more communities or groups in MLA that they felt that they belonged to. Sixty-nine percent strongly agreed or agreed that they were able to find at least 1 community or group in which to belong; 10% strongly disagreed or disagreed; and 21% neither agreed nor disagreed.

#### Positive professional growth.

There was a question of whether or not respondents agreed with the statement: “My experience in MLA has had a positive influence on my professional growth.” Seventy-six percent strongly agreed or agreed with the statement; 6% strongly disagreed or disagreed; and 18% neither agreed nor disagreed.

### Demographics

There were multiple questions designed to capture demographic information from the membership. Questions varied from “Where do you work” and “How long have you been in the profession” to more inclusive questions about gender, sexuality, and religious identities.

#### Age.

**Figure 2:**
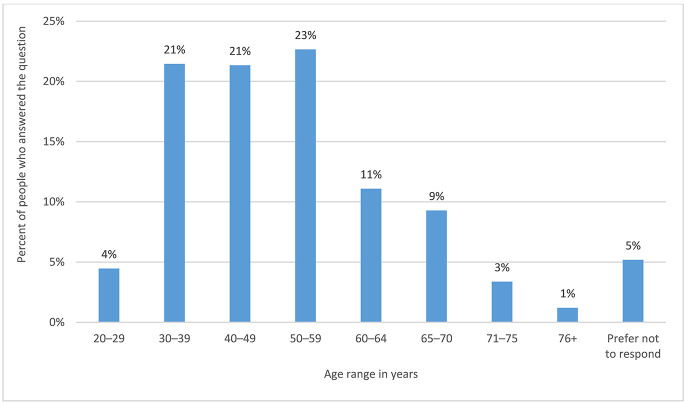
Age range by percentage

The respondents had a main age range of 30–39 (21%), 40–49 (21%), and 50–59 (23%). Figure 2 shows the full age range distribution.

#### Race and ethnicity.

**Figure 3:**
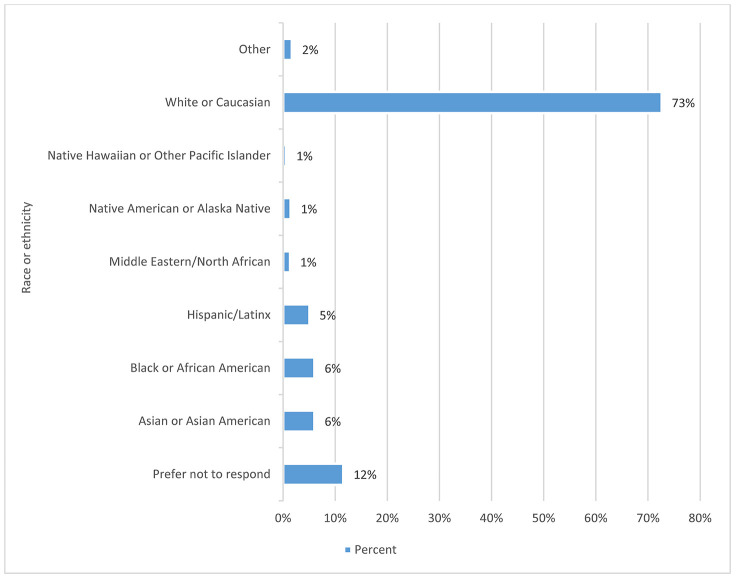
Race/ethnicity by percentage

The largest group was “white or Caucasian” at 73%. Figure 3 provides a further breakdown in distribution. There were 14 comments received from this question, most of which focused on more specific groups, like immigrant, South Asian, and Indo-Caribbean.

#### Country of residence for work or school.

Ninety-five percent of respondents marked that they resided in the United States for work or school. Two percent indicated that they worked or learned in Canada, and 2% marked outside of the United States or Canada. The other 2% of respondents indicated that they were from all over the world, including Africa, Europe, Australia, the Caribbean, South America, and the Middle East.

#### Military status.

Ninety-seven percent of respondents to the survey indicated that they had never served in the US military; 2% indicated that they were veterans; and 0.3% indicated that they were currently serving.

#### Gender identity.

Seventy-nine percent indicated female, and 13% indicated male. One percent marked gender queer, and 0.1% each indicated non-binary, transgender female, and transgender male, respectively. Of the 4 comments received for this question, 2 of them were negative toward the question and 2 further elucidated their gender identity.

#### Sexual orientation.

**Figure 4:**
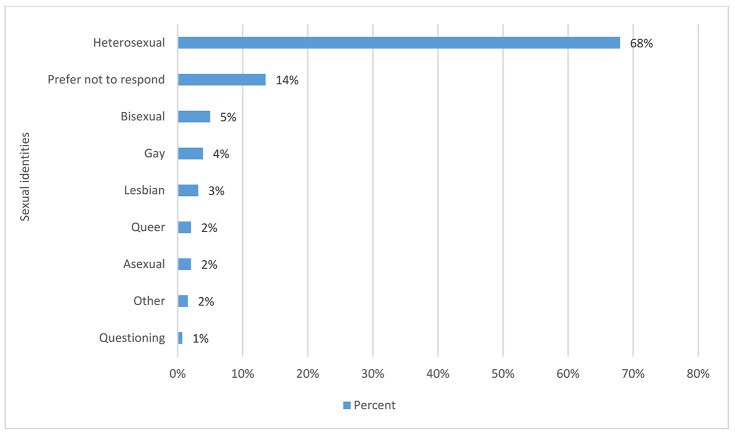
Sexual identity by percentage

Sixty-eight percent of people marked heterosexual as their sexual orientation. The rest of respondents fell across the spectrum of responses in the single digits, with 13% preferring not to respond to the question. Figure 4 shows the full range of responses to this question. The range of comments with this question indicated that some respondents were offended by being asked about their sexual orientation. Also, some comments showed that some respondents did not understand that the terms “straight” and “heterosexual” are synonymous.

#### Ability.

**Figure 5:**
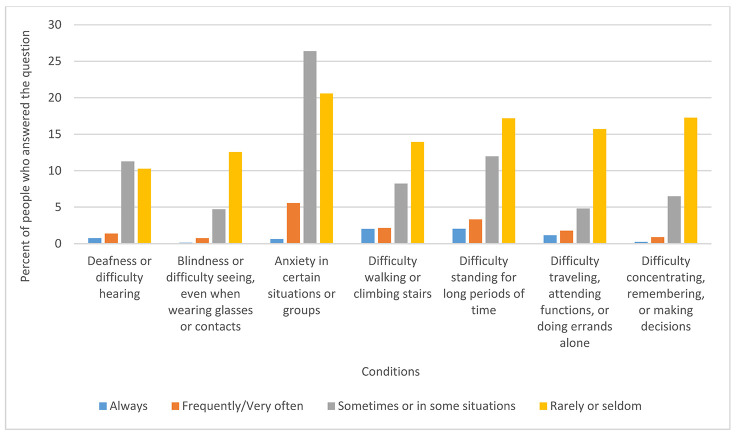
Ability by percentage

While the bulk of respondents answered “not applicable” in terms of ability, the fact that so many people responded to the question at all is groundbreaking and indicates that there are more librarians with disabilities in the profession than believed to be in the past. Twenty-six percent of respondents marked that they had anxiety sometimes or in some situations. Figure 5 shows a full breakdown of responses to this question. There were thirty-five comments associated with this question, of which the topics ranged from being “not applicable” to the question, to discussions of environmental concerns at MLA annual meetings, to naming specific disabilities that respondents had.

#### Religion.

**Figure 6:**
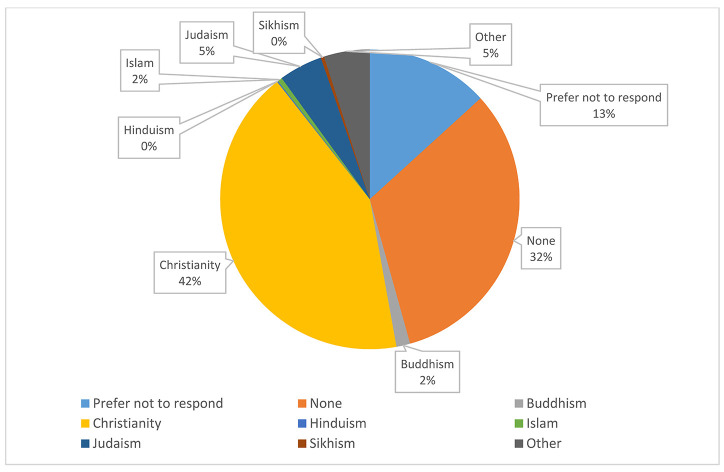
Religion by percentage

While the bulk of respondents identified as Christian at 42%, a large percentage of respondents marked “none” for this question at 32%. There were 40 comments with this question of which many delineated specific sects of Christianity, such as Catholicism. Figure 6 shows the variety of religions represented.

Please note that in Figure 6 that Hinduism is at 0.1% and Sikhism is at 0.4%.

There was a follow-up qualitative question regarding religion that asked, “How, if at all, should MLA make allowance for individual religious considerations when planning events, meetings, or other activities?” A total of 437 comments responded to this question. After coding responses, of which there might be more than 1 code per comment, 188 comments indicated that there should be some allowance for religious considerations, and this most often took the suggested form of MLA providing some space for prayer or meditation and/or providing a list of local religious institutions if members wanted to visit them for religious observance. One hundred seventy-three comments focused on not scheduling the meeting during major holy days. Eighty-eight comments mentioned food options in some way, including kosher and halal options. Fifty-five responses indicated that the association should not take religion into consideration at all, and 28 comments were neutral, had no opinion, or were suggestions unrelated to the question. Finally, 19 responses asked that Sunday mornings be kept unscheduled so that members can follow religious observances.

#### Caregiving.

**Figure 7:**
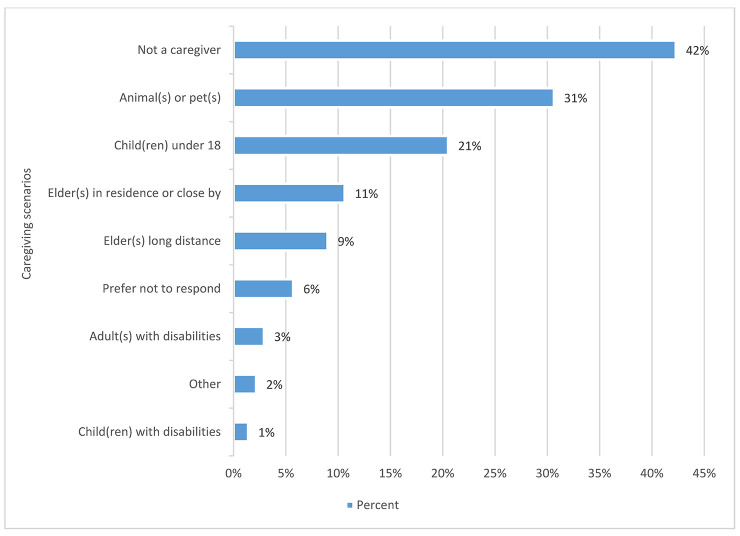
Caregiving by percentage

Forty-two percent of respondents indicated that they were not caregivers in anyway. This indicated that the bulk of those who answered the question were, in fact, caregivers. Figure 7 shows the complete data range to this question. Seventeen comments were associated with this question, many of which indicated who or how the respondent cared for others, including long distance caring. Several comments indicated displeasure at the question.

#### Education.

The members of the association who took the survey were extremely well educated. However, discrepancies in the data suggested that many respondents did not accurately interpret the meaning of the question, “Please indicate the degree(s) you have earned.” Ninety-three percent indicated they had a master's degree in library and information science, but only 58% indicated they had a bachelor's degree. Since a bachelor's degree is usually a prerequisite to postgraduate degrees, the data for this question are suspect. There were 39 comments for this question, most of which focused on types of degrees or mentioned different types of certificates achieved.

#### Financial aid.

**Figure 8:**
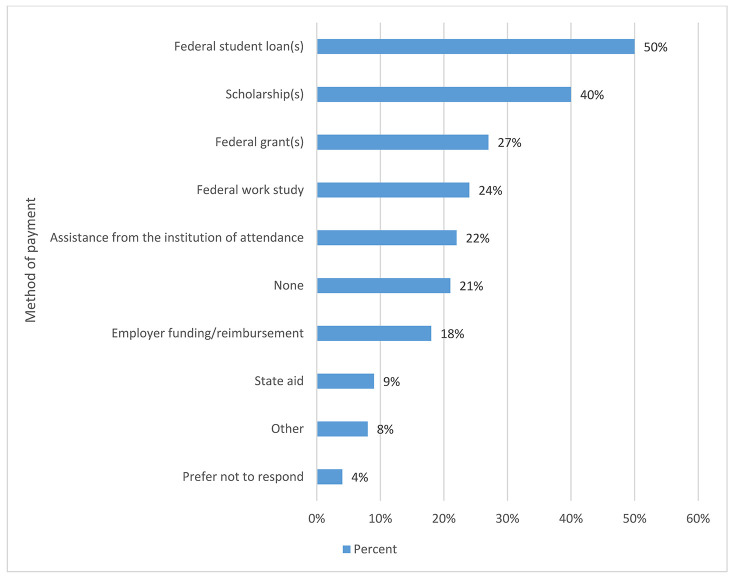
Financial aid by percentage

This question, in part, helped answer the question the task force had on the socioeconomic status of the membership. Most respondents (50%) took out federal student loans for their educations. Figure 8 provides the full distribution of responses. There were 61 comments with this question, most of which focused on combinations of how the respondents funded their education, including private loans and the GI Bill.

#### Primary employment status.

Survey respondents were asked what their current employment status was. Ninety percent indicated that they were employed full time; 4% indicated part time; 4% stated retired; 0.7% stated they were students or not currently employed; and 0.6% marked other. There were 5 comments with this question, of which all of the responses fell under the above-mentioned categories.

#### Current job function.

There were 25 options for respondents to choose from when selecting what their job role was. The largest selection was for medical librarian/librarian at 21%. Table 1 lists all 25 options and the percentages of response. There were also 39 comments with this question, most of which indicated selections that could have been made within the 25 options that were available.

**Table 1:** Job function by percentage

Job function	Percent
Medical librarian/librarian	21%
Director of the health sciences/medical library	14%
Reference/information services	10%
Department or division head	7%
Embedded/liaison/outreach librarian or informationist	6%
One-person or solo librarian with multiple responsibilities	6%
Other	5%
Assistant/associate director	4%
Clinical librarian	4%
Education/instructional services	4%
Research or data services	3%
Retired	3%
Subject specialist librarian	3%
Collection management/technical services	2%
Electronic resources librarian	2%
Consumer health	1%
Library support staff/paraprofessional	0.9%
Access/circulation services librarian	0.6%
Emerging technologies librarian	0.6%
Self-employed or consultant	0.5%
Student	0.5%
Systems/computer services	0.5%
Public librarian	0.3%
Vendor representative	0.3%
Web/Internet services	0.3%

#### Pay range.

**Figure 9:**
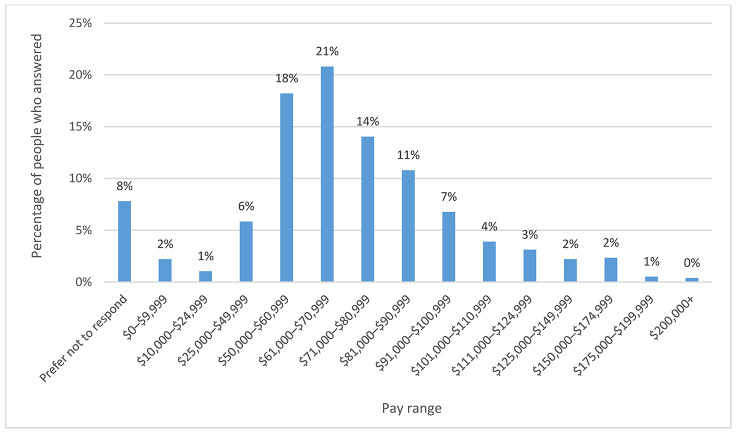
Pay range by percentage

The results of the pay range question had a cluster between $50,000–$80,999, of which the highest percentage was 21% between $61,000–$70,999. Figure 9 shows the complete range of responses to this question.

## CONCLUSION

This survey represented the first opportunity for MLA to survey the membership regarding substantial demographics that included ability, gender, sexuality, and ethnicity. The results of the survey represent a snapshot in time of the membership that took the survey. It is the hope of the Diversity and Inclusion Task Force that this membership survey will be repeated every three years in order to build a demographic history of the association and how it changes over time.

## SUPPLEMENTAL FILE

**Appendix:** Survey

