# Supplementary material for: Medical Library Association Diversity and Inclusion Task Force 2019 Survey Report
Source: J Med Libr Assoc. 2020 Jul 1;108(3):503–12. doi: 10.5195/jmla.2020.948 (PMC7441896; doi:10.5195/jmla.2020.948)
Supplement: Supplementary file 1 — Appendix: Survey [file jmla-108-3-503-s01.pdf]

JMLA Journal of the Medical Library Association

SC

Supplemental content to
J Med Libr Assoc. Jul;108(3):dx.doi.org/10.5195/jmla.2020.948
www.jmla.mlanet.org
© Pionke 2020

Medical Library Association Diversity and Inclusion Task Force 2019 Survey Report

JJ Pionke, MA, MSI

APPENDIX

Survey

1. Are you a current member of the Medical Library Association (MLA)?
Yes, No

2. For how many years have you been a member of MLA?
0–4 years, 5–9 years, 10–14 years, 15–19 years, 20–24 years, 25 years or more

3. What is your current primary employment status?
Full time, Part time, Retired, Student, Not currently employed, Other

4. I feel valued as an individual in MLA.
Strongly agree, Agree, Neither agree nor disagree, Disagree, Strongly disagree

5. I feel a sense of belong in MLA.
Strongly agree, Agree, Neither agree nor disagree, Disagree, Strongly disagree

6. I feel MLA has a strong commitment to diversity, equity, and inclusion.
Strongly agree, Agree, Neither agree nor disagree, Disagree, Strongly disagree

7. I feel I am treated with respect in MLA.
Strongly agree, Agree, Neither agree nor disagree, Disagree, Strongly disagree

8. If you do not feel respected in MLA, would you please tell us why?
[Open response]

9. In MLA, I have the opportunity to participate at the level I desire.
Strongly agree, Agree, Neither agree nor disagree, Disagree, Strongly disagree

10. If you have not had the opportunity to participate in MLA at your desired level, please provide us with additional details.
[Open response]

11. I have found one or more MLA communities or groups where I feel I belong.
Strongly agree, Agree, Neither agree nor disagree, Disagree, Strongly disagree

12. My experience in MLA has had a positive influence on my professional growth.
Strongly agree, Agree, Neither agree nor disagree, Disagree, Strongly disagree

13. I have felt welcomed and included at MLA annual meetings.
Strongly agree, Agree, Neither agree nor disagree, Disagree, Strongly disagree

Supplemental content to
J Med Libr Assoc. Jul;108(3):dx.doi.org/10.5195/jmla.2020.948
www.jmla.mlanet.org
© Pionke 2020

14. If you have not felt welcome or included at an MLA annual meeting, please provide further detail.
[Open response]

15. How would you characterize the amount of emphasis MLA places on issues of diversity, equity, and inclusion.
Far too little, Too little, About right, Too much, Far too much

16. In relation to diversity, equity, and inclusion, please rate your satisfaction with the overall MLA environment that you have experienced since becoming a member.
Very satisfied, Satisfied, Neither satisfied nor dissatisfied, Dissatisfied, Very dissatisfied

17. What is your current age (range)?
Prefer not to respond, 20–29 years, 30–39 years, 40–49 years, 50–59 years, 60–64 years, 65–70 years, 71–75 years, 76 years or more

18. Please indicate the racial OR ethnic groups with which you identify (select all that apply):
Prefer not to respond, Asian or Asian American, Black or African American, Hispanic/Latinx, Middle Eastern/North African, Native American or Alaskan Native, Native Hawaiian or Other Pacific Islander, White or Caucasian, Other group (please specify)

19. In which country do you currently live and work or go to school?
United States, Canada, Outside the United States or Canada

20. Have you ever served in the United States Armed Forces, Military Reserves, or National Guard?
I am currently serving, I have served and am no longer serving, I have never served

21. What is your gender/gender identity?
Prefer not to respond, Female, Gender Queer, Male, Non-Binary, Transgender female, Transgender male, Other response not listed

22. What is your sexual orientation?
Prefer not to respond, Asexual, Bisexual, Gay, Heterosexual, Lesbian, Queer, Questioning, Other response not listed

23. Please indicate if any of the following conditions currently limited your participation in MLA; please add clarifying or additional information in the comments box if you wish to:
Deafness or difficulty hearing; Blindness or have difficulty seeing, even when wearing glasses or contacts; Anxiety in certain situations or groups; Difficulty walking or climbing stairs; Difficulty standing for long periods of time; Difficulty traveling, attending functions, or doing errands alone; Difficulty concentrating, remembering, or making decisions

24. With what religion, if any, do you most identify?
Prefer not to respond, None, Buddhism, Christianity, Hinduism, Islam, Judaism, Sikhism, Another religion (please specify)

25. How, if at all, should MLA make allowance for individual religious considerations when planning events, meetings, or other activities?
[Open response]

JMLA Journal of the Medical Library Association

SC
Supplemental content to
J Med Libr Assoc. Jul;108(3):dx.doi.org/10.5195/jmla.2020.948
www.jmla.mlanet.org
© Pionke 2020

26. If you participate in caregiving, please indicate whom you take care of (select only those that apply):
Prefer not to respond, I am not a caregiver, Child(ren) under age 18, Child(ren) with disabilities, Adult(s) with disabilities, Elder(s) in residence or close by, Elder(s) long distance, Animal(s) or pet(s), Other (please specify)

27. Please indicate the degree(s) you have earned (select all that apply):
Two-year college degree (associate's), Four-year college degree (bachelor's), Master's degree in the arts and sciences (MA, MS), Master's degree in library or information science (MLS, MLIS), Professional master's degree (e.g., MBA, MPH, MSW, MPA, MSE, MSN, MFA), PhD, Other doctoral degree (e.g., EdD, DDiv, DrPH, DBA), Medical degree (MD, DO, DDS, DVM), Law degree (JD, LLM, SJD), Other (please specify)

28. What, if any, financial aid for college did you receive? Please select all that apply:
Prefer not to respond, None, Federal grant(s), Federal work-study, Federal student loan(s), State aid, Assistance from the institution I attended, Scholarship(s), Employer funding/tuition reimbursement, Other (please specify)

29. Please indicate your primary job function or current role (select only one):
Access/circulation services librarian, Assistant/associate director, Clinical librarian, Collection management/technical services, Consumer health, Department or division head, Director of the health sciences/medical library, Education/instructional services, Electronic resources librarian, Embedded/liaison/outreach librarian or informationist, Emerging technologies librarian, Library support staff/paraprofessional, Medical librarian/librarian, One-person or solo librarian with multiple responsibilities, Public librarian, Reference/information services, Research or data services, Retired, Self-employed or consultant, Student, Subject specialist librarian, Systems/computing services, Vendor representative, Web/Internet services, Other (please specify)

30. What is the pay range of your annual salary (exclude any benefits value OR nonlibrary employment)?
Prefer not to respond, $0–$9,999, $10,000–$24,999, $25,000–$49,999, $50,000–$60,999, $61,000–$70,999, $71,000–$80,999, $81,000–$90,999, $91,000–$100,999, $101,000–$110,999, $111,000–$124,999, $125,000–$149,999, $150,000–$174,999, $175,000–$199,999, $200,000 or more

31. Does your employer pay for your annual MLA membership?
Yes, full membership fee outside of any professional development funds; Yes, full membership fee if I choose to use professional development for membership; Yes, part of my membership (e.g., offers the $50 rebate option); No; Other (please specify)
